# Ventilator-Associated Pneumonia Prediction Models Based on AI: Scoping Review

**DOI:** 10.2196/57026

**Published:** 2024-05-14

**Authors:** Jinbo Zhang, Pingping Yang, Lu Zeng, Shan Li, Jiamei Zhou

**Affiliations:** 1 Nursing Department, Affiliated Hospital of Zunyi Medical University, Zunyi, China; 2 Nursing College, Zunyi Medical University, Zunyi, China

**Keywords:** artificial intelligence, machine learning, ventilator-associated pneumonia, prediction, scoping, PRISMA, Preferred Reporting Items for Systematic Reviews and Meta-Analyses

## Abstract

**Background:**

Ventilator-associated pneumonia (VAP) is a serious complication of mechanical ventilation therapy that affects patients’ treatments and prognoses. Owing to its excellent data mining capabilities, artificial intelligence (AI) has been increasingly used to predict VAP.

**Objective:**

This paper reviews VAP prediction models that are based on AI, providing a reference for the early identification of high-risk groups in future clinical practice.

**Methods:**

A scoping review was conducted in accordance with the PRISMA-ScR (Preferred Reporting Items for Systematic Reviews and Meta-Analyses extension for Scoping Reviews) guidelines. The Wanfang database, the Chinese Biomedical Literature Database, Cochrane Library, Web of Science, PubMed, MEDLINE, and Embase were searched to identify relevant articles. Study selection and data extraction were independently conducted by 2 reviewers. The data extracted from the included studies were synthesized narratively.

**Results:**

Of the 137 publications retrieved, 11 were included in this scoping review. The included studies reported the use of AI for predicting VAP. All 11 studies predicted VAP occurrence, and studies on VAP prognosis were excluded. Further, these studies used text data, and none of them involved imaging data. Public databases were the primary sources of data for model building (studies: 6/11, 55%), and 5 studies had sample sizes of <1000. Machine learning was the primary algorithm for studying the VAP prediction models. However, deep learning and large language models were not used to construct VAP prediction models. The random forest model was the most commonly used model (studies: 5/11, 45%). All studies only performed internal validations, and none of them addressed how to implement and apply the final model in real-life clinical settings.

**Conclusions:**

This review presents an overview of studies that used AI to predict and diagnose VAP. AI models have better predictive performance than traditional methods and are expected to provide indispensable tools for VAP risk prediction in the future. However, the current research is in the model construction and validation stage, and the implementation of and guidance for clinical VAP prediction require further research.

## Introduction

### Background

Ventilator-associated pneumonia (VAP) is a pulmonary infectious disease that occurs in patients who receive mechanical ventilation for more than 48 hours and is primarily caused by pathogens that are present in the hospital environment. VAP is one of the most common complications in patients who undergo invasive mechanical ventilation. The incidence of VAP among patients who undergo mechanical ventilation ranges from 5% to 40%, depending on the setting and diagnostic criteria. The estimated attributable mortality rate of VAP is approximately 10%, with higher mortality rates among surgical intensive care unit (ICU) patients and those with moderate severity scores at admission [1]. VAP seriously affects the treatments and prognoses of patients, resulting in prolonged hospital stays, increased medical costs, and increased mortality rates. The early identification of groups at high risk for VAP is important for reducing VAP incidence and mortality [2].

Artificial intelligence (AI) can contribute to significant developments in the medical field. With the popularity of electronic health records, advancements in hardware computing power, and the development of big data, AI has become the optimal tool [3]. Among predictive models, AI models perform better than traditional models in various ways [4]. Data mining of patient cases via AI technology is conducted to create tools that can predict groups at high risk for VAP to help medical staff initiate preventive interventions early, which is critical for reducing VAP incidence and mortality. Therefore, we aimed to explore the application of AI technology in predicting VAP and report our findings to provide a reference for the future development of VAP prevention.

### Research Problem and Objective

Many studies have been conducted on the application of AI to VAP prediction. However, there is a lack of integrated evidence describing the AI techniques and model features that have been used in existing research. Therefore, this review aims to explore the characteristics of AI models for VAP prediction to assist the scientific community in advancing research within this field by identifying gaps and planning for the future.

## Methods

### Overview

We conducted a scoping review of studies that used AI to predict and diagnose VAP. For a transparent review, the guidelines of the PRISMA-ScR (Preferred Reporting Items for Systematic Reviews and Meta-Analyses extension for Scoping Reviews) [5] were followed.

### Search Strategy

The following seven literature databases were searched for this study: the Wanfang database, the Chinese Biomedical Literature Database, Cochrane Library, Web of Science, PubMed, MEDLINE, and Embase. Databases were searched by using terms related to the target technology, population, and outcomes of interest. The search queries used for each database are listed in Table 1. In addition to searching the databases, backward citation screening was performed on the included studies to identify additional relevant studies. The search was conducted from January 12 to January 16, 2024.

**Table 1.** Search terms used to find studies.

Database	Hits, n	Search terms
Wanfang database	3	*(“Ventilator-associated pneumonia” OR “ventilator-associated pneumonia” OR “ventilator-associated pneumonia”) AND (“Prediction” OR “predictive models” OR “risk prediction” OR “assessment” OR “risk assessment tools”) AND (“Artificial intelligence” OR “machine learning” OR “artificial learning” OR “deep learning” OR “Bayesian learning” OR “neural networks” OR “support vector machines” OR “statistical learning” OR “decision trees” OR “random forests”)* (in Chinese)
Chinese Biomedical Literature Database	1	*(“Ventilator-associated pneumonia” OR “ventilator-associated pneumonia” OR “ventilator-associated pneumonia”) AND (“Prediction” OR “predictive models” OR “risk prediction” OR “assessment” OR “risk assessment tools”) AND (“Artificial intelligence” OR “machine learning” OR “artificial learning” OR “deep learning” OR “Bayesian learning” OR “neural networks” OR “support vector machines” OR “statistical learning” OR “decision trees” OR “random forests”)* (in Chinese)
Cochrane Library	10	(“vap” OR “Pneumonia Ventilator-Associated” OR “Ventilator-Associated Pneumonia”) AND (“Prediction” OR “prediction model” OR “risk prediction” OR “assessment” OR” risk assessment” OR “assessment tool”) AND (“artificial intelligence” OR “machine learning” OR “Artificial Learning” OR “deep learning” OR “Bayesian Learning” OR “Neural Network” OR “Support vector machine” OR “Statistical Learning” OR “Decision tree*” OR “Random Forest”)
Web of Science	29	(“vap” OR “Pneumonia Ventilator-Associated” OR “Ventilator-Associated Pneumonia”) AND (“Prediction” OR “prediction model” OR “risk prediction” OR “assessment” OR” risk assessment” OR “assessment tool”) AND (“artificial intelligence” OR “machine learning” OR “Artificial Learning” OR “deep learning” OR “Bayesian Learning” OR “Neural Network” OR “Support vector machine” OR “Statistical Learning” OR “Decision tree*” OR “Random Forest”)
PubMed	45	(“vap” OR “Pneumonia Ventilator-Associated” OR “Ventilator-Associated Pneumonia”) AND (“Prediction” OR “prediction model” OR “risk prediction” OR “assessment” OR” risk assessment” OR “assessment tool”) AND (“artificial intelligence” OR “machine learning” OR “Artificial Learning” OR “deep learning” OR “Bayesian Learning” OR “Neural Network” OR “Support vector machine” OR “Statistical Learning” OR “Decision tree*” OR “Random Forest”)
MEDLINE	21	(“vap” OR “Pneumonia Ventilator-Associated” OR “Ventilator-Associated Pneumonia”) AND (“Prediction” OR “prediction model” OR “risk prediction” OR “assessment” OR” risk assessment” OR “assessment tool”) AND (“artificial intelligence” OR “machine learning” OR “Artificial Learning” OR “deep learning” OR “Bayesian Learning” OR “Neural Network” OR “Support vector machine” OR “Statistical Learning” OR “Decision tree*” OR “Random Forest”)
Embase	28	(“vap” OR “Pneumonia Ventilator-Associated” OR “Ventilator-Associated Pneumonia”) AND (“Prediction” OR “prediction model” OR “risk prediction” OR “assessment” OR” risk assessment” OR “assessment tool”) AND (“artificial intelligence” OR “machine learning” OR “Artificial Learning” OR “deep learning” OR “Bayesian Learning” OR “Neural Network” OR “Support vector machine” OR “Statistical Learning” OR “Decision tree*” OR “Random Forest”)

### Eligibility Criteria

This review included studies on AI technology for VAP diagnosis and risk prediction. However, this review excluded literature reviews and other articles that only summarized AI approaches to VAP analysis and studies that were based solely on clinical trials and experimental studies. We included only journal articles and conference papers and excluded case reports, reviews, white papers, conference abstracts, editorials, and gray literature. Studies that used non-AI techniques to predict VAP were excluded. Moreover, this review considered only studies that were written in English and Chinese and were published between the date of the establishment of the repository and January 2024. There were no constraints with regard to the study settings, study designs, study outcomes, publication months, or publication countries.

### Study Selection

The screening process was performed by 2 researchers. First, we imported document titles into EndNote (Clarivate) software to eliminate duplicates. As per the inclusion criteria, irrelevant articles were further excluded by reading the titles and abstracts. Subsequently, the full texts were read to determine the final included articles. Any objections during screening were discussed with a third investigator.

### Data Extraction and Synthesis

Two reviewers independently extracted the data from the included literature and discussed them with a third reviewer in cases of any objections. The extracted information included the authors; year of publication; study design; country; sample source; study population; sample size; positive outcomes; tool type; construction method; main evaluation content; model presentation form; verification method; and indicators related to reliability, validity, and predictive power.

Narrative synthesis was used to analyze the extracted data. The results included in this study were categorized as technical characteristics of the included studies (eg, AI models and algorithms used), AI model data (eg, data sources), and predictive performance indices.

### Ethical Considerations

This study did not require ethical approval because we did not study any human or animal subjects and did not collect any personal information or sensitive data.

## Results

### Search Results

**Figure 1.**
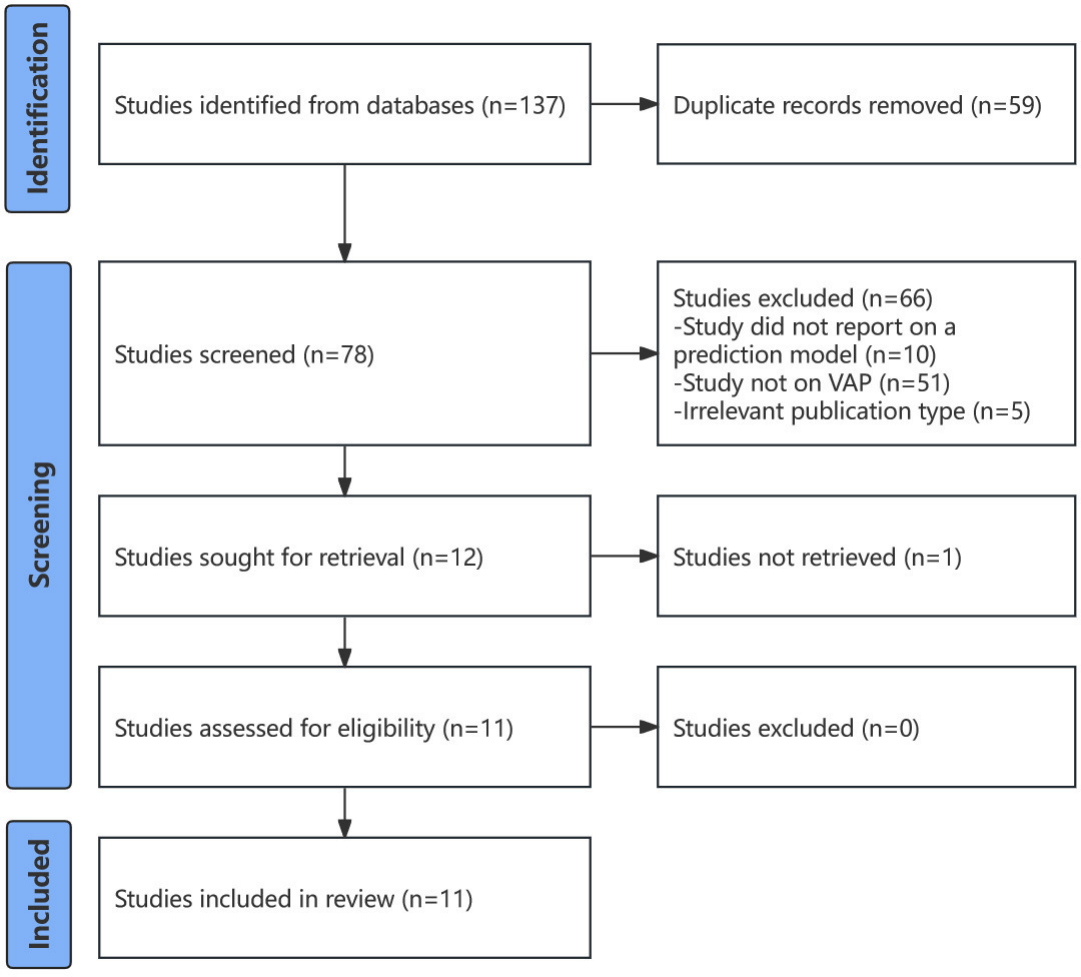
Flow diagram of the review process and the identification of studies via databases. VAP: ventilator-associated pneumonia.

As shown in Figure 1, 137 studies were retrieved from the search, and 59 were duplicates. A total of 78 study titles and abstracts were screened, and 66 were excluded. Figure 1 presents the reasons for exclusion. Because the full text of 1 study could not be found, 11 studies were screened for eligibility; all of them met the criteria and were included in this review.

### Characteristics of Included Studies

All included studies (11/11, 100%) were published in peer-reviewed journals. The studies were published between 2007 and 2023 (Table 1), with most (3/11, 27%) published in 2023. The included studies were from 4 countries but were predominantly from the United States (5/11, 45%), followed by China (4/11, 36%). In addition, ICU patients were the most frequently studied population (studies: 6/11, 55%), 2 studies involved neurosurgical ICU patients, 1 study involved patients with traumatic brain injury, 1 study involved pediatric ICU patients, and 1 study involved older patients (age≥65 y). Public databases were the most common sources of samples (studies: 6/11, 55%), with 4 studies using the MIMIC-III (Medical Information Mart for Intensive Care III) data set. The detailed characteristics of the included studies are summarized in Table 2.

**Table 2.** Characteristics of the included studies (N=11).

Author, year	Publication type	Study design	Country	Sample source	Study population
Schurink et al [6], 2007	Journal article	Prospective cohort study	Netherlands	Recruit volunteers	Medical ICU^a^ and neurosurgical ICU patients
Rambaud et al [7], 2023	Journal article	Retrospective cohort study	France	Electronic medical records	PICU^b^ patients
Pearl and Bar-Or [8], 2012	Journal article	Retrospective cohort study	United States	NTDB^c^ data set 6.2	ICU patients
Chen et al [9], 2020	Journal article	Prospective case-control study	China	Recruit volunteers	ICU patients
Liang et al [10], 2022	Journal article	Retrospective cohort study	China	MIMIC-III^d^ data set	ICU patients
Faucher et al [11], 2022	Preprint article	Retrospective cohort study	United States	MIMIC-III data set	ICU patients
Liao et al [12], 2019	Journal article	Prospective case-control study	China	Recruit volunteers	Neurosurgical ICU patients
Abujaber et al [13], 2021	Journal article	Retrospective cohort study	United States	Electronic medical records	Patients with traumatic brain injury
Giang et al [14], 2021	Journal article	Retrospective cohort study	United States	MIMIC-III data set	ICU patients
Samadani et al [15], 2023	Journal article	Retrospective case-control study	United States	Philips eRI^e^ data set	ICU patients
Mingwei et al [16], 2023	Journal article	Retrospective cohort study	China	MIMIC-III data set	Older patients (aged ≥65 y)

a ICU: intensive care unit.

b PICU: pediatric intensive care unit.

c NTDB: National Trauma Data Bank.

d MIMIC-III: Medical Information Mart for Intensive Care III.

e eRI: eICU Research Institute.

### AI Technical Characteristics of Included Studies

All 11 included studies used only machine learning algorithms, and none of them involved deep learning algorithms or large language models. The random forest model was the most commonly used model (studies: 5/11, 45%), followed by the XGBoost (extreme gradient boost) model (studies: 4/11, 36%) and neural networks (studies: 3/11, 27%). Only 4 studies mentioned the programming languages for model building (Python: 3/11, 27%; R: 1/11, 9%). Further, 3 studies used model-building software to develop predictive models (ie, Hugin, Tiberius, and SPSS Modeler 18.2). Further details are presented in Table 3.

**Table 3.** Basic characteristics, predictors, and performance of artificial intelligence models for ventilator-associated pneumonia prediction (studies: N=11).

Author	Model	Development methodology	Sample size, n	Positive outcome, n	Predictors	Application	Verification method	Prediction performance
Schurink et al [6]	BDSS^a^	Hugin	872	157	Body temperature: <36.5 °C or >38.5 °C; ICU^b^ daily sputum score: none=+0, rarely=+1, moderate=+2, severe=+3; sputum score: >14; sputum color: yellow or green; PaO_2_^c^/FiO_2_^d^: ≤205 mm Hg or decrease of >35 mm Hg from the previous day; use of acetaminophen, nonsteroidal anti-inflammatory drugs, or steroid antipyretics; chest x-ray showing localized or diffuse infiltration of the lungs; WBC^e^ count: <4×10^9^/L or >11×10^9^/L; MV^f^ time: >48 h	—^g^	Not reported	AUC^h^: 0.846 (95% CI 0.794-0.899); sensitivity: 0.79; specificity: 0.79; positive predictive value: 0.87; negative predictive value: 0.66
Rambaud et al [7]	IRF^i^	R	827	77	Body weight (kg); WBC count (per mm^3^); neutrophil count (per mm^3^); PaO_2_ (mm Hg); FiO_2_ (%); PEEP^j^ (cmH_2_O); PIP^k^ (cmH_2_O); MAwP^l^ (cmH_2_O); respiratory rate (respirations per min); tidal volume (mL); subjective volume of respiratory secretions (0, +, ++, and +++); lung dynamic compliance calculated by the oxygenation index and oxygen saturation index (in barometric mode: tidal volume/[PIP – PEEP]; in volumetric mode: tidal volume/[peak pressure – PEEP]); PIM^m^ 2 score; PELOD-2^n^ score	—	k-fold cross-validation	AUC: 0.82 (95% CI 0.71-0.93); sensitivity: 0.797; specificity: 0.727; positive predictive value: 0.09; negative predictive rate: 0.99; accuracy: 0.795
Pearl and Bar-Or [8]	ANN^o^	Tiberius	1,438,035	598,066	ICU length of stay; trauma score (ISS^p^); no ventilation; gender; systolic blood pressure: <40 mm Hg; age: ≤16 y; respiratory rate: <10 respirations per minute; respiratory rate: >29 respirations per minute; full model; age: >55 y	—	Not reported	Gini coefficient: 0.80435
Chen et al [9]	KNN^q^, NBM^r^, DT^s^, NN^t^, SVM^u^, and RF^v^	Python	59	26	Electronic nose sensor data	—	Not reported	Best model—AUC: 0.94 (95% CI 0.74-1.00); accuracy: 0.77 (95% CI 0.46-0.95); sensitivity: 0.71; specificity: 0.83; positive predictive value: 0.93; negative predictive rate: 0.71
Liang et al [10]	RF	Python	10,431	212	Internal intensive care (control: other intensive care); emergency admission; hypertension; liver failure; PaO_2_/FiO_2_; APACHE^w^ III score; temperature; respiratory rate; A-aDO_2_^x^/PaO_2_; urinary output; blood sodium; bilirubin; GCS^y^; SOFA^z^; pulmonary function; coagulation function; liver function; cardiovascular disease; central nervous system disease; aspiration admission; trauma admission	—	Not reported	AUC: mean 0.84 (SD 0.02); sensitivity: mean 0.74 (SD 0.03); specificity: mean 0.71 (SD 0.01)
Faucher et al [11]	LR^aa^, fEBM^ab^, and XGBoost^ac^	—	18,671	470	WBC first; WBC mean; MV hours (value); WBC median; WBC last; GCS last; WBC max; WBC min; GCS median; GCS mean; GCS max; RespRate^ad^ first; Dias ABP^ae^ max; blood (count) × MV hours (value); MV hours (value) × WBC last; MV hours (value) × WBC first; weight; weight × MV hours (value); SpO_2_^af^ first; MV hours (value) × WBC median	—	Not reported	Best model (fEBM)—AUC: 0.893
Liao et al [12]	ENN^ag^ and SVM	—	12	12	Electronic nose sensor data	—	Not reported	ENN—accuracy: mean 0.9479 (SD 0.0135); sensitivity: mean 0.9714 (SD 0.0131); positive predictive value: mean 0.9288 (SD 0.0306); AUC: mean 0.9842 (SD 0.0058). SVM—accuracy: mean 0.8686 (SD 0.0422); sensitivity: mean 0.9250 (SD 0.0423); positive predictive value: mean 0.8639 (SD 0.0276); AUC: mean 0.9410 (SD 0.0301)
Abujaber et al [13]	DT	SPSS Modeler 18.2	772	169	Time to emergency department; blood transfusion; ISS^p^; pneumothorax; comorbidity	—	Not reported	Accuracy: 0.835; AUC: 0.805; precision: 0.71; negative predicted value: 0.86; sensitivity: 0.43; specificity: 0.95; *F*-score: 0.54
Giang et al [14]	LR, MLP^ah^, RF, and XGBoost	—	6126	524	MV hours; biotics indicator; sputum indicator; sputum count; GCS_LAST; Platelets_MIN; Platelets_MAX; Platelets_AVERAGE; blood culture count; Temp_FIRST; GCS_AVERAGE; Platelets_FIRST; GCS_MAX; Platelets_MEDIAN; WBC_LAST	—	Not reported	Best model—AUC: 0.854
Samadani et al [15]	XGBoost	—	14,923	6811	Body temperature; FiO_2_; age; MV times; total CO_2_^ai^; chloride; SpO_2_; heart rate; respiratory rate; gender; PaCO_2_^aj^; creatinine; BUN^ak^; mean blood pressure; hematocrit	—	Hold-out cross-validation	AUC: 0.76; AUPRC^al^: 0.75
Mingwei et al [16]	LR, RF, XGBoost, and LightGBM^am^	Python	1523	336	SOFA; maximum WBC count; maximum respiratory rate; maximum base remaining; age; maximum creatinine; minimum PaCO_2_; minimum oxygenation index; diabetes; ICU admission, paraplegia, gender, COPD^an^	—	10-fold cross-validation	Best models: LightGBM—AUC: 0.85 (95% CI 0.82-0.88); accuracy: 0.77; precision: 0.80; recall: 0.72; specificity: 0.82; *F*_1_: 0.75. XGBoost—AUC: 0.84 (95% CI 0.81-0.87); accuracy: 0.76; precision: 0.78; recall: 0.73; specificity: 0.79; *F*_1_:0.75

a BDSS: Bayesian decision support system.

b ICU: intensive care unit.

c PaO_2_: partial pressure of oxygen.

d FiO_2_: fraction of inspired oxygen.

e WBC: white blood cell.

f MV: mechanical ventilation.

g Not applicable.

h AUC: area under the curve.

i IRF: imbalanced random forest model.

j PEEP: positive end-expiratory pressure.

k PIP: peak inspiratory pressure.

l MAwP: mean airway pressure.

m PIM: pediatric index of mortality.

n PELOD-2: Pediatric Logistic Organ Dysfunction-2.

o ANN: artificial neural network.

p ISS: Injury Severity Score.

q KNN: k-nearest neighbor.

r NBM: naive Bayes model.

s DT: decision tree.

t NN: neural network.

u SVM: support vector machine.

v RF: random forest.

w APACHE: Acute Physiology and Chronic Health Evaluation.

x A-aDO2: alveolar-arterial oxygen difference.

y GCS: Glasgow Coma Scale.

z SOFA: Sequential Organ Failure Assessment.

aa LR: logistic regression.

ab fEBM: full feature explainable boosting machine.

ac XGBoost: extreme gradient boost.

ad RespRate: respiratory rate of the ventilator.

ae Dias ABP: diastolic blood pressure.

af SpO_2_: peripheral blood oxygen saturation.

ag ENN: ensemble neural network.

ah MLP: multilayer perceptron.

ai CO_2_: carbon dioxide.

aj PaCO_2_: carbon dioxide partial pressure.

ak BUN: blood urea nitrogen.

al AUPRC: area under the precision-recall curve.

am LightGBM: light gradient boosting machine.

an COPD: chronic obstructive pulmonary disease.

Different types of data were used in the included studies, including laboratory data (eg, white blood cell count, neutrophil count, and bilirubin level), clinical data (including temperature, sputum volume, and ventilator parameters), and demographic data (eg, age, weight, and sex). Of note, 2 studies used sensor data to build predictive models, and the remaining 9 studies used clinical data. In addition, 67% (6/9) of these studies used laboratory data, with white blood cell count being the most commonly used laboratory data (studies: 4/9, 44%), followed by neutrophil count (studies: 1/9, 11%), bilirubin level (studies: 1/9, 11%), and blood urea nitrogen level (studies: 1/9, 11%). Demographic data were used in 56% (5/9) of the studies; age was used as a predictor in 4 studies, and weight and age were both included in only 1 study.

In terms of data set size, of the 11 studies, 6 (55%) had sample sizes of >1000; however, with regard to the data from the electronic nose sensors that were used in 2 studies, multiple sensors were placed on the electronic nose, and each sensor collected data more than once. Therefore, the actual sample sizes for these two studies were 1888 [9] and 3360 [12]. Nevertheless, because the data were collected by the same electronic nose sensor and came from the same patient, we did not include these two studies in the number of studies with sample sizes of >1000. Further, 3 studies used data sets with <1000 samples, and 4 studies had data sets with >10,000 samples. The AI performance index was mentioned in all 11 studies. The area under the curve (AUC) was the most commonly used predictive performance index (studies: 10/11, 90%), followed by sensitivity (studies: 6/11, 55%) and specificity (studies: 6/11, 55%). The AUC values, which were reported in 10 studies, averaged to 0.86 (SD 0.07) and ranged from 0.76 to 0.98. The sensitivity, which was reported in 6 studies, averaged to 0.74 (SD 0.18) and ranged from 0.43 to 0.97. The specificity, which was reported in 6 studies, averaged to 0.80 (SD 0.09) and ranged from 0.71 to 0.95. Additionally, 5 studies reported accuracy (mean 0.82, SD 0.07, range 0.77-0.95).

## Discussion

### Principal Findings

In this review, we explored AI techniques for the prediction of VAP. Of the 11 included studies, 9 (82%) were published in the past 5 years, and the number of studies has increased annually with the evolution of AI technology (1 in 2019, 1 in 2020, 2 in 2021, 2 in 2022, and 3 in 2023). Most (9/11, 82%) of the AI-based prediction model studies were published in the United States (5/11, 45%) and China (4/11, 36%). To explore the application of AI in predicting VAP, the results were divided into 3 categories, and each of them classified the included studies from a different perspective.

The first category included the technical characteristics of the studies. All studies used only machine learning algorithms, with the random forest model being the most commonly used model (studies: 5/11, 45%), followed by neural networks (studies: 4/11, 36%) and the XGBoost model (studies: 4/11, 36%). The second category focused on AI model data, in which we explored the data types, data sources, and data set sizes. Different types of data, including laboratory, clinical, and demographic data, were used in the included studies. In terms of data set size, apart from 2 studies that used electronic noses, 6 (55%) had sample sizes of >1000. Public databases were the most common sources of data (studies: 6/11, 55%). The third category focused on the predictive performance of AI models, including studies that used different performance validation indices, such as the AUC, accuracy, sensitivity, and specificity.

### Implications for Practice and Research

This review highlights the most common AI models that have been used to predict VAP. Based on our findings, AI models can predict VAP by using various data types. In our review, no studies that used deep learning and large language models were found. A possible reason for this is that chest computed tomography data are not available in most public databases, and in clinical practice, patients who do not exhibit pneumonia symptoms do not undergo chest computed tomography examinations; therefore, such data are not available for research. The random forest and XGBoost models are the most frequently used machine learning–based VAP prediction models, probably because ensemble learning models exhibit better prediction performance and robustness when dealing with multiple types of data compared to other models [17].

Based on the data sources of the prediction models, the use of more data types for comprehensive predictions may be the main focus of future research. Current research may be constrained to using structured data, owing to the limitations of algorithms and data collection workloads, while electronic health records contain unstructured clinical text, such as admission records and progress notes. Furthermore, much data remain to be mined. Tsai et al [18] found that information extracted from unstructured clinical text could make predictive models more comprehensive and improve their predictive performance. In addition to unstructured clinical text, lung radiography and computed tomography can be used to predict the occurrence of pneumonia.

In terms of predictive tools, natural language processing and deep learning may be the direction of future research, and the development of large language models, such as ChatGPT, that are based on natural language processing is sufficient to prove the ability of natural language processing algorithms to process unstructured clinical text [19]. Traditional machine learning algorithms are not competent in the image recognition domain, while deep learning algorithms can analyze and process clinical imaging data effectively. Lee et al [20] found that deep learning–based predictive models that used preoperative imaging data from patients could effectively predict the occurrence of postoperative pneumonia; however, no studies have used deep learning algorithms to construct VAP prediction models.

Of further note, the studies reviewed herein rarely mentioned nurse-related data, and it has been suggested that nursing is important for VAP prevention [21,22]. The potential of various data types in predicting VAP should be explored in future studies. Additionally, none of the studies included in this review considered the application of the final model. The deployment of feasible predictive models in clinical settings needs to be explored.

### Strengths

This review discusses all of the AI techniques and study populations that have been used to date to predict VAP, with no major restrictions on paper status, research environment, and geographic location. In addition, the characteristics of each AI model and the data sets that were used to build the models were discussed in depth.

Based on our findings, Frondelius et al [4,23] explored diagnostic and prognostic models for VAP and performed a meta-analysis of the performance of machine learning–based predictive models for VAP. However, to the best of our knowledge, ours is the first review of all AI VAP prediction models that have been explored thus far, filling research gaps to improve understanding of prediction techniques rather than focusing solely on the final predictive performance of models. Moreover, in the literature search, we did not place any limitations on types of technology and included all branches of AI to gain insight into the research on different AI technologies for VAP prediction.

Finally, study selection and data extraction were performed independently by 2 evaluators to ensure minimal bias.

### Limitations

This review has certain limitations. Reviews, conference abstracts, case reports, white papers, proposals, editorials, and gray literature were excluded to reduce the complexity of the results. We also included Chinese databases in our search but did not explore articles in languages other than English or Chinese, which might have reduced the comprehensiveness of our study.

### Conclusions

This paper reviews the application of AI technology in VAP prediction and provides new evidence on the role of AI technology. We believe that the findings will help researchers better understand the application of AI technology in VAP prediction and provide a reference for future research on VAP prediction models. Lastly, we believe that advances in AI technology will provide further possibilities for predicting VAP and that interdisciplinary developments will improve the health care industry.

## Supplementary material

10.2196/57026 Checklist 1 PRISMA-ScR (Preferred Reporting Items for Systematic Reviews and Meta-Analyses extension for Scoping Reviews) checklist.

## Abbreviations

AI: artificial intelligence
AUC: area under the curve
ICU: intensive care unit
MIMIC-III: Medical Information Mart for Intensive Care III
PRISMA-ScR: Preferred Reporting Items for Systematic Reviews and Meta-Analyses extension for Scoping Reviews
VAP: ventilator-associated pneumonia
XGBoost: extreme gradient boost
